# Environmental Enrichment, Age, and PPARα Interact to Regulate Proliferation in Neurogenic Niches

**DOI:** 10.3389/fnins.2016.00089

**Published:** 2016-03-09

**Authors:** Margarita Pérez-Martín, Patricia Rivera, Eduardo Blanco, Clara Lorefice, Juan Decara, Francisco J. Pavón, Antonia Serrano, Fernando Rodríguez de Fonseca, Juan Suárez

**Affiliations:** ^1^Departamento de Biología Celular, Genética y Fisiología, Instituto de Investigación Biomédica de Málaga, Universidad de MálagaMálaga, Spain; ^2^UGC Salud Mental, Instituto de Investigación Biomédica de Málaga, Universidad de Málaga-Hospital Universitario Regional de MálagaMálaga, Spain; ^3^Departament de Pedagogia i Psicologia, Facultat d'Educació, Psicologia i Treball Social, Universitat de LleidaLleida, Spain

**Keywords:** aging, environment, PPARα, subventricular zone, hippocampus, neurogenesis

## Abstract

Peroxisome proliferator-activated receptor alpha (PPARα) ligands have been shown to modulate recovery after brain insults such as ischemia and irradiation by enhancing neurogenesis. In the present study, we investigated the effect of the genetic deletion of PPARα receptors on the proliferative rate of neural precursor cells (NPC) in the adult brain. The study was performed in aged *Ppar*α^−/−^ mice exposed to nutritional (treats) and environmental (games) enrichments for 20 days. We performed immunohistochemical analyses of cells containing the replicating cell DNA marker 5-bromo-2′-deoxyuridine (BrdU+) and the immature neuronal marker doublecortin (Dcx+) in the main neurogenic zones of the adult brain: subgranular zone of dentate gyrus (SGZ), subventricular zone of lateral ventricles (SVZ), and/or hypothalamus. Results indicated a reduction in the number of BrdU+ cells in the neurogenic zones analyzed as well as Dcx+ cells in the SGZ during aging (2, 6, and 18 months). *Ppar*α deficiency alleviated the age-related reduction of NPC proliferation (BrdU+ cells) in the SVZ of the 18-months-old mice. While no genotype effect on NPC proliferation was detected in the SGZ during aging, an accentuated reduction in the number of Dcx+ cells was observed in the SGZ of the 6-months-old *Ppar*α^−/−^ mice. Exposing the 18-months-old mice to nutritional and environmental enrichments reversed the *Ppar*α^−/−^-induced impairment of NPC proliferation in the neurogenic zones analyzed. The enriched environment did not modify the number of SGZ Dcx+ cells in the 18 months old *Ppar*α^−/−^ mice. These results identify PPARα receptors as a potential target to counteract the naturally observed decline in adult NPC proliferation associated with aging and impoverished environments.

## Introduction

Neural progenitor/proliferative cells (NPC) are derived from embryonic radial-glial cells that have the ability to divide, self-renew and generate functional differentiated cells (neurons and glia) during the entire life of the animal (Doetsch, 2003b). NPC are localized in discrete regions of the adult mammalian brain called stem cells niches, such as the subventricular zone (SVZ) lining the walls of the lateral ventricles and the subgranular zone (SGZ) of the hippocampal dentate gyrus, which are capable of promoting neurogenesis and gliogenesis (Doetsch et al., 1999; Doetsch, 2003a; Lie et al., 2004; Albayram et al., 2011). In the last decade, several studies evidenced the hypothalamus as a novel neurogenic zone in the adult brain, especially after insults (Rivera et al., 2011; Robins et al., 2013; Maggi et al., 2014; Lin and Iacovitti, 2015; Lin et al., 2015).

It is well-known that NPC proliferation and neuronal differentiation are regulated by intrinsic (growth factors, neurotransmitters, hormones) and external (environment, caloric intake, drugs) factors, as well as by epigenetic mechanisms that intermediate in between the environment and the genome (Alvarez-Buylla and Lim, 2004; Arias-Carrion et al., 2007; Montalban-Loro et al., 2015). A critical factor affecting the rate of adult neurogenesis is age (Drapeau et al., 2003; Aizawa et al., 2009; Coras et al., 2010; Ngwenya et al., 2015). In adult rodents, hippocampal neurogenesis persists throughout the lifespan but suffers from progressive age-associated declines (Kuhn et al., 1996; McDonald and Wojtowicz, 2005). This fact is relevant since aging is also associated with an increased risk for cerebral insults, even in healthy subjects. Multiple studies have linked the age-induced decline of NPC and neurogenesis with neurodegenerative diseases and cognitive performance (Van Praag et al., 2005; Paillard, 2015). However, others reports have suggested that such a relationship does not exist (Bizon and Gallagher, 2003; Merrill et al., 2003). Several factors have a positive influence on cerebral decline associated with normal aging. In this line, environmental enrichment, including nutrition and physical activity, may improve brain function in normal animals and in animals with brain-related disorders, such as Alzheimer and other aging-related cerebral diseases, which, in part, are likely mediated through the enhancement of neurogenesis (Fan et al., 2007; Zhao et al., 2015; Garthe et al., 2016).

Peroxisome proliferator-activated receptors (PPARs) belong to a nuclear receptor superfamily capable of regulating physiological responses associated with inflammatory responses, energy metabolism, and cell proliferation, differentiation, migration and survival (Rosen and Spiegelman, 2001; Smith et al., 2003; Fidaleo et al., 2014). It has been reported that PPARα and its endogenous ligands (eicosanoids, leukotrienes, and endocannabinoid-like molecules such as oleoylethanolamide or OEA) support a role in neuroprotection against oxidative stress, which is target for neurodegenerative diseases and contribute to normal brain aging (Nunomura et al., 2012; Zolezzi et al., 2013; Fidaleo et al., 2014). PPARα is expressed in neuronal, astroglial, and ependymal cells and may be relevant in glutamatergic, dopaminergic, and cholinergic neurotransmission (Zhou and Waxman, 1998; Avshalumov and Rice, 2002; Melis et al., 2013). For instance, PPARα activity modulates acetylcholine release and ameliorates cognitive and memory decline associated with aging (Hajjar et al., 2012). OEA-PPARα interaction facilitates memory consolidation through noradrenergic activity (Campolongo et al., 2009), modulates satiety responses through hypothalamic neurons (Romano et al., 2013), and regulates motivational responses for alcohol through the peripheral nervous system (Bilbao et al., 2015). Interestingly, the involvement of PPARα in cell proliferation and apoptosis (Roberts et al., 2002; Cimini et al., 2007; Cimini and Ceru, 2008) as well as neural cell differentiation and maturation has been demonstrated (Cristiano et al., 2005; Bento-Abreu et al., 2007; Fandel et al., 2013). PPARα activation (for instance, through the elevation of the PPARα endogenous ligands in the brain) preserves hippocampal neurogenesis and inhibits microglial activity (Ramanan et al., 2009; Rivera et al., 2015a).

Although, PPARα may be a potential therapeutic target in neurodegenerative, neuroinflammatory and neurocognitive alterations related to Alzheimer and Parkinson's diseases (Plaza-Zabala et al., 2010; Scuderi et al., 2012; Fidaleo et al., 2014; González-Aparicio et al., 2014), the involvement of PPARα in age-related decline and environmental enrichment-induced enhancement of adult neurogenesis is still uncertain. The present study designed an experimental approach to investigate the potential role of PPARα in adult NPC proliferation by focusing on the impact of sustained external stimulation through food and play. Immunohistochemistry were performed to analyze cells that contained the replicating cell DNA marker 5-bromo-2′-deoxyuridine (BrdU+) in the main neurogenic zones (SVZ, SGZ, and hypothalamus), as well as cells expressing the immature neuronal factor doublecortin (Dcx+) in the SGZ. This study was performed in adult aged mice lacking *Ppar*α gene expression (*Ppar*α^−/−^) that were previously exposed to nutritional (treats) and environmental (games) enrichments for 20 days.

## Materials and methods

### Ethics statement

The protocols and procedures were approved by the Ethics Committee of Malaga University (CEUMA: 2014-0001-A) and performed in compliance with European animal research laws [European Communities Council Directives2010/63/UE, 90/219/CEE, Regulation (EC) No 1946/2003] and Spanish National and Regional Guidelines for Animal Experimentation and Use of Genetically Modified Organisms (Real Decreto 53/2013, Ley 32/2007, and Ley 9/2003, Real Decreto 178/2004, Decreto 320/2010). All efforts were made to minimize animal suffering and reduce the number of animals used.

### Animals

Adult wild-type (WT) and *Ppar*α^−/−^ (KO) male mice (The Jackson Laboratories, Bar Harbor, ME, USA) derived from intercrosses between heterozygous *Ppar*α^+/−^ mice on a C57Bl/6 background were jointly housed in cages and maintained in standard conditions at 20 ± 2°C room temperature with 40 ± 5% relative humidity and a 12-h light/dark cycle with a dawn/dusk effect (Animal House, University of Málaga). Standard rodent chow (Prolab RMH 2500, 2.9 kcal/g) and water were available *ad libitum*.

### Aging

The SVZ, SGZ and hypothalamus of the WT and *Ppar*α^−/−^ mice at ages of 2, 6, and 18 months old were analyzed. Six experimental groups were obtained depending on the age and genotype (*n* = 6 mice per group): WT 2 months group, WT 6 months group, WT 18 months group, *Ppar*α^−/−^ (KO) 2 months group, *Ppar*α^−/−^ (KO) 6 months group, and *Ppar*α^−/−^ (KO) 18 months group.

### Environmental enrichment

**Figure 1:**
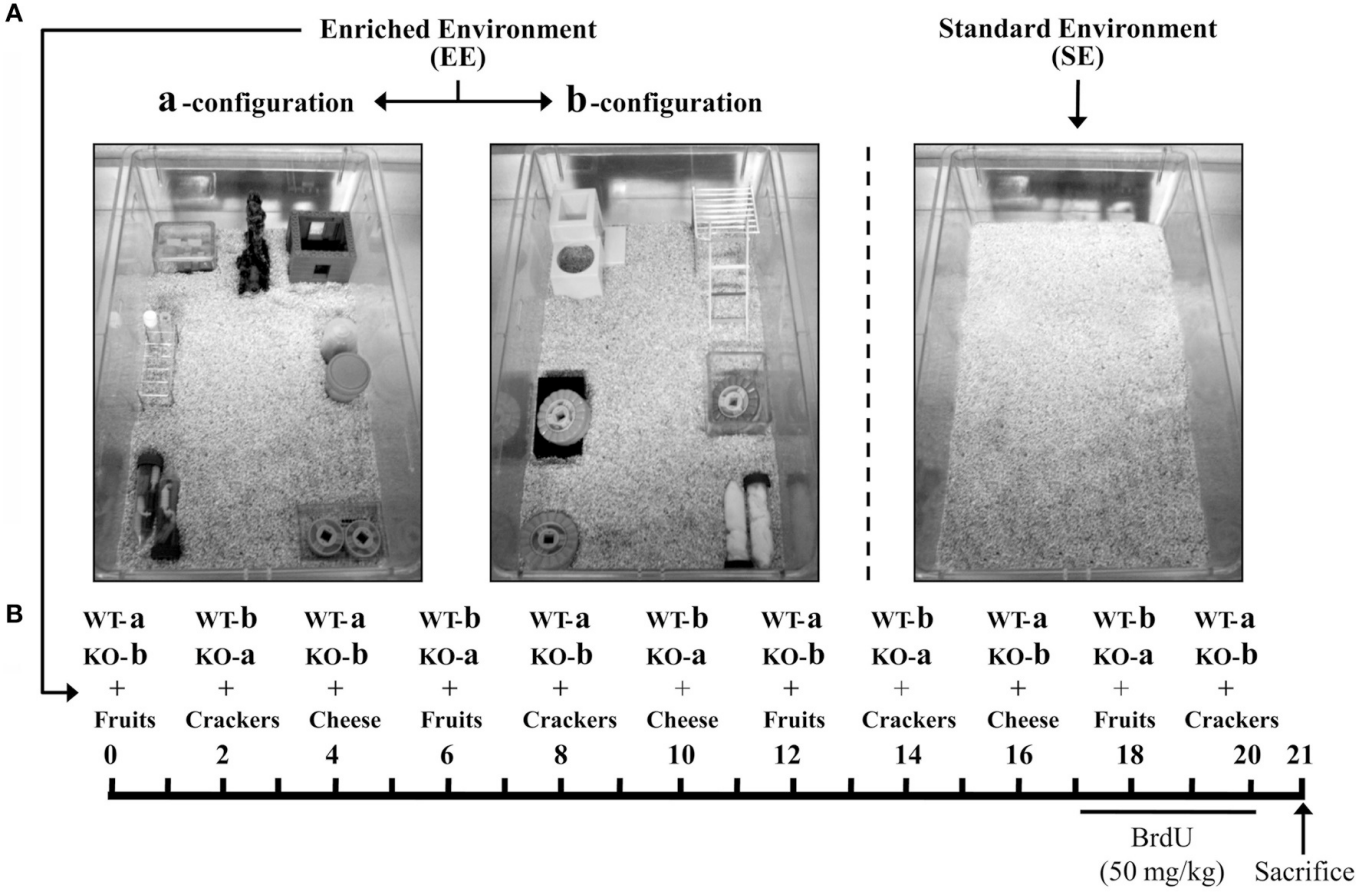
**(A)** Housing conditions of the wild-type (WT) and *Ppar*α^−/−^ (KO) mice. The photographs illustrated the different set of environmental configurations: two enriched environmental configurations (a and b) consisting of a contextual stimulation with ramps, floor platforms, tunnels and toys, and a standard environment (a standard housing cage). **(B)** Schematic diagram showing the experiment design. The mice from each genotype were randomly assigned to the standard environment (SE) or the nutritional and environmental enrichment (EE) for 20 days. The mice rotated between the enriched configurations a and b every 2 days. Nutritional enrichment consisted of the administration of different treats such as fruits, crackers, and cheese in cycles of 6 days. The last 4 days previous to sacrifice, all animals were injected with BrdU (50 mg/kg).

A new batch of WT and *Ppar*α^−/−^ mice at 18 months old (*n* = 10 animals per genotype) were randomly selected to perform the environmental enrichment experiments. Thus, half of the mice from each genotype (*n* = 5) were housed in an enriched environment (EE) or standard environment (SE) cages for 20 days (Figure 1). We designed two EE configurations (a and b, see Figure 1A), which consisted of two large cages (59.5 × 38 × 20 cm) with different set of objects and games such as ramps, floor platforms, tunnels, and toys. WT and *Ppar*α^−/−^ mice were changed to a or b environmental configurations every 2 days, up to 20 days, to avoid habituation induced by prolonged contextual (spatial) stimulation. Nutritional enrichment was also performed by adding different treats such as fruits, crackers, and cheese in cycles of 6 days for 20 days (Figure 1B). The control WT and *Ppar*α^−/−^ mice were housed in standard environment cages (equal cage dimension but no objects inside) and fed with the standard chow but without nutritional enrichment (treats). Four experimental groups were obtained depending on the genotype and environment (*n* = 5 mice per group): WT SE group, WT EE group, *Ppar*α^−/−^ (KO) SE group, and *Ppar*α^−/−^ (KO) EE group.

### BrdU administration

5′-bromo-2′-deoxyuridine (BrdU, cat. no. B5002, Sigma, St. Louis, MO, USA) was dissolved at 15 mg/mL in sterile 0.9% NaCl solution. BrdU was i.p. administered at a dose of 50 mg/kg body weight once per day for 3 consecutive days (day 17–19) and twice at the last EE-day (day 20, 4 h between injections). The animals were killed 12 h after the last injection of BrdU was administered.

### Brain collection

All animals were intraperitoneally (i.p.) anesthetized (sodium pentobarbital, 50 mg/kg body weight) and transcardially perfused with 4% formaldehyde in 0.1 M phosphate buffer (PB). The brains were dissected out and kept in the same fixative solution overnight at 4°C. The brains were then cryoprotected and cut into 30-μm-thick coronal sections by using a sliding microtome (Leica VT1000S). Sections were divided in eight parallel series until use for immunohistochemistry.

### Immunohistochemistry

Free-floating coronal sections from −1.58 to −2.46 mm Bregma levels (hippocampus and hypothalamus) and 1.42 to −0.10 mm Bregma levels (striatum) from one of the five parallel series obtained from each mouse brain were selected for each immunohistochemistry (Paxinos and Franklin, 2004). Sections were first washed several times with PBS to remove sodium azide. Then, sections were incubated in a solution of 3% hydrogen peroxide and 10% methanol in PB 0.1 M for 45 min at room temperature in darkness to inactivate endogenous peroxidase. After three washes in PBS for 10 min, DNA was denatured by exposing sections to HCl 2 N for 15 min at 37°C, followed by two washes in borate buffer 0.15 M to neutralize pH. After three additional washes in PBS for 10 min, sections were incubated in a blocking solution containing 0.3% albumin, 0.3% triton X-100 and 0.05% sodium azide in PBS for 45 min. Sections were incubated overnight in the primary antibody rat anti-BrdU (1:500; Accurate Chemical & Scientific, Westbury, NY, USA, cat. no. OBT0030G) at 4°C. For doublecortin immunohistochemistry, selected sections at hippocampal levels were firstly incubated in a solution of 3% hydrogen peroxide and 10% methanol in PB 0.1 M for 10 min, secondly incubated in a blocking solution containing 5% horse serum, 0.3% triton X-100 and 0.05% sodium azide in PBS for 1 h, and finally incubated overnight in the primary antibody goat anti-doublecortin (1:500; Santa Cruz Biotechnology, cat. no. sc-8066) at room temperature. The following day the sections were incubated in the biotinylated donkey anti-rat IgG (H+L) antibody (1:500, Novex; cat. no. A18743) or the biotinylated horse anti-goat IgG antibody (1:1000, Vector; cat. no. BA-9500) for 90 min. The sections were then incubated in ExtrAvidin peroxidase (Sigma, St. Louis, MO) diluted 1:2000 in darkness at room temperature for 1 h. Finally, immunolabeling was revealed with 0.05% diaminobenzidine (DAB; Sigma) and 0.03% H_2_O_2_ in PBS.

### Quantification of BrdU and doublecortin-immunoreactive cells

The average density of positive cells per animal was quantified. Thus, the estimation of the number of cells per section (30 μm deep) and area (mm^2^) in both hemispheres was calculated according to the following formula: Na = ∑(*Q*-)/∑(*a*_*str*_), where ∑*Q*- is the total number of positive cells counted per animal and *a*_*str*_ is the area of the structure analyzed. Each structure analyzed consisted of ~6 coronal sections, which resulted in one of every five equidistant sections (one representative section for each 180 μm) according to the rostro-caudal extent. To outline the area of study, the region of interest was drawn in each structure, whose identification was performed at Bregma −1.58 to −2.46 mm in hippocampal and hypothalamic levels, and at Bregma 1.42 to −0.10 mm in striatal levels according to a mouse brain atlas and cytoarchitectonic criteria (Paxinos and Franklin, 2004). Thus, the BrdU-immunoreactive (-ir) nuclei and doublecortin-ir cells that came into focus were manually counted in the subgranular zone (SGZ) of the dentate gyrus and/or the subventricular zone (SVZ) of the lateral ventricles. Regarding the hypothalamus, counting was performed in the ventromedial (VMH) and arcuate (ARC) nuclei of the hypothalamus and median eminence. Quantification were performed using a standard optical microscope with the 40X objective (Nikon Instruments Europe B.V., Amstelveen, The Netherlands) coupled to the NIS-Elements Imaging Software 3.00 (Nikon). The data were expressed as the number of positive cells per area (mm^2^).

### Statistical analysis

Statistical analysis of the results was performed using the computer program GraphPad Prism version 5.04 (GraphPad Software Inc., San Diego, CA, USA). Data were represented as the mean ± s.e.m. for five-six determinations depending on the experimental group. Statistical analysis was performed using two-way ANOVA followed by Bonferroni *post hoc* test for multiple comparisons. *P* < 0.05 was considered to be significant.

## Results

### Effect of age and genotype on SVZ, SGZ, and hypothalamic cell proliferation

**Figure 2:**
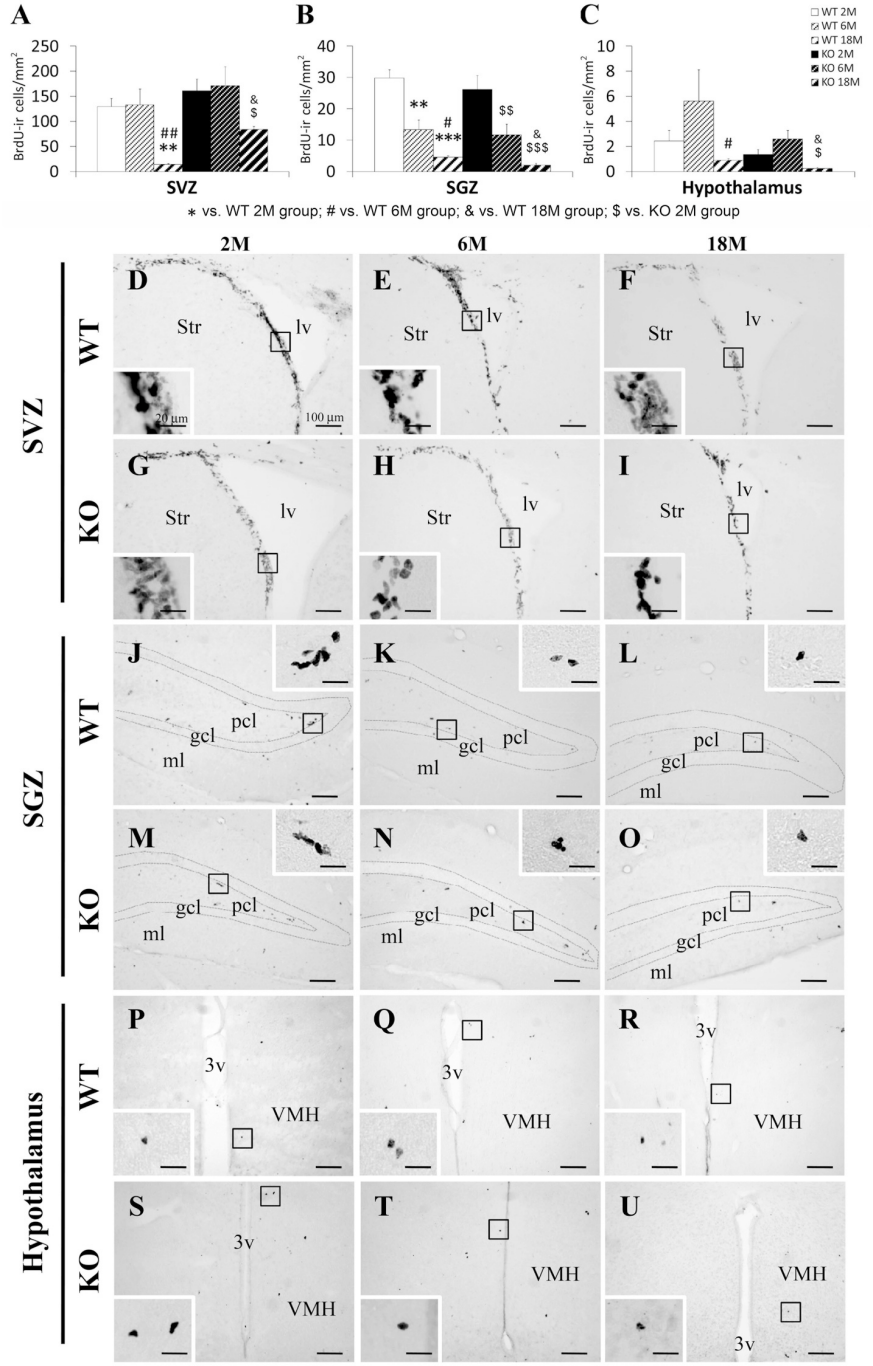
**Effect of age (2, 6, and 18 months) on the number of cells that contained BrdU in the SVZ (A), SGZ (B), and hypothalamus (C) of WT and *Ppar*α^−/−^ (KO) mice**. The data were expressed as the media of the number of BrdU+ cells per area (mm^2^) + s.e.m (*N* = 6). Bonferroni's test: ^**^P < 0.01, ^***^p < 0.001 vs. WT 2M group; ^#^*P* < 0.05, ^*##*^*P* < 0.01 vs. WT 6M group; ^&^*P* < 0.05 vs. WT 18M group; ^$^*P* < 0.05, ^$$^*P* < 0.01, ^$$$^*P* < 0.001 vs. KO 2M group. **(D–U)** Representative photomicrographs showing low and high (insets) magnification views of the typical clustering of newborn cells containing the BrdU labeling in the SGZ, SVZ, and hypothalamus of 2, 6, and 18 months old WT and KO mice. Scale bars (100 μm and 20 μm for insets) are included in each image.

Typical clustering of newborn cells containing nuclear BrdU labeling was observed in the SVZ of the lateral ventricles extending from the ventral to the dorsolateral ventricular peak and into the rostral migratory stream following a transversal view of the adult mouse brain (Figure 2). This arrangement of the SVZ BrdU+ cells was more evident in the younger mice that the older ones. Scattered BrdU+ cells were found in the SGZ of the dentate gyrus as well as in a hypothalamic area, by the wall of the third ventricle, including the hypothalamic ventromedial and arcuate nuclei, and the median eminence. Qualitatively, we observed a less number of cells containing BrdU in the three neurogenic niches during the ages analyzed (Figure 2).

Two-way ANOVA showed an age effect (2, 6, and 18 months) on the three neurogenic areas [SVZ: *F*_(2, 19)_ = 12.98; *P* < 0.001; SGZ: *F*_(2, 19)_ = 41.73, *P* < 0.001; hypothalamus: *F*_(2, 19)_ = 5.51; *P* < 0.05; Figure 2]. Interestingly, a genotype effect (WT and KO) was found in the number of BrdU+ cells in the SVZ [*F*_(1, 19)_ = 6.45, *P* < 0.05], but not in the SGZ or hypothalamus. No interaction between age and genotype was detected, that is, PPARα deficiency did not produce a different effect on cell proliferation during aging.

After performing the Bonferroni analysis, we found a decreased number of BrdU+ cells in the SVZ of the lateral ventricles of the 18 months old WT mice compared to that of the 2 and 6 months old WT mice (^**##^*P* < 0.01; (Figures 2A,D–F). The number of BrdU+ cells was lower in the SVZ of the 18 months old KO mice than that of the 6 months old KO mice (^$^*P* < 0.05), but in increased compared to those of the 18 months old WT mice (^&^*P* < 0.05; (Figures 2A,F–I). In the SGZ of the dentate gyrus, the number of BrdU+ cells decreased in the 6 and 18 months old WT mice (^***^P < 0.01 and ^***^P < 0.001, respectively) and the 6 and 18 months old KO mice (^$$^*P* < 0.01 and ^$$$^*P* < 0.001, respectively) compared to the respective 2 months old WT and KO mice (Figures 2B,J–O). The number of BrdU+ cells in the SGZ of the 18 months old WT mice was also decreased when it was compared to that of the 6 months old WT mice (^#^*P* < 0.05; Figures 2B,K,L). The number of BrdU+ cells in the SGZ of the 18 months old KO mice was decreased when it was compared to that of the 18 months old WT mice (^&^*P* < 0.05; Figures 2B,L,O). A decrease of BrdU+ cells was also found in the hypothalamus of the 18 months old WT and KO mice compared to the respective 6 months old WT and KO mice (^$^*P* < 0.05; Figures 2C,Q,R,T,U). The number of BrdU+ cells in the hypothalamus of the 18 months old KO mice showed a decrease when it was compared to that of the 18 months old WT mice (^&^*P* < 0.05; Figures 2C,R,U).

### Effect of environmental enrichment and genotype on SVZ, SGZ, and hypothalamic cell proliferation in 18 months old mice

**Figure 3:**
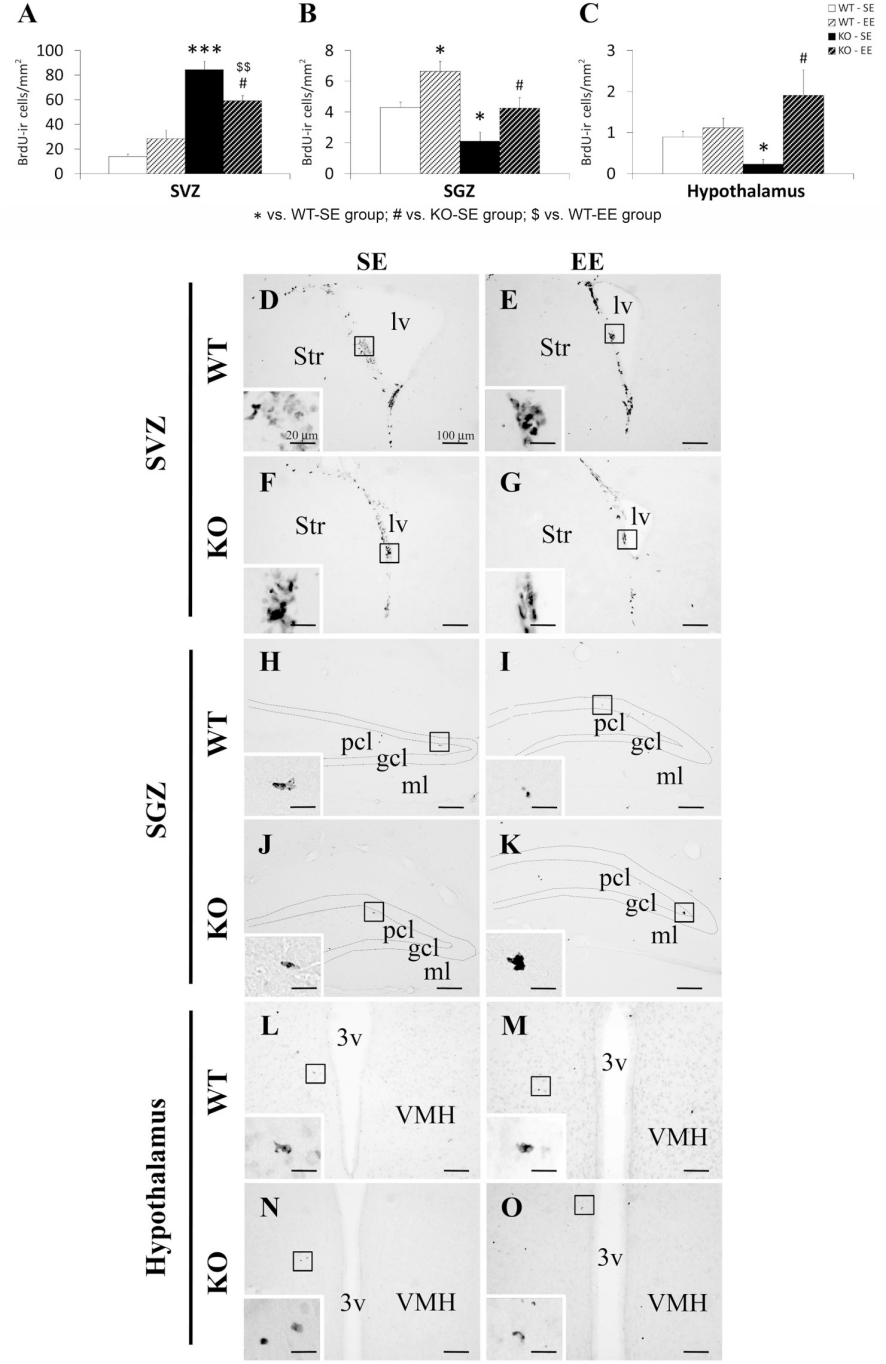
**Effect of environment (SE and EE) on the number of cells that contained BrdU in the SVZ (A), SGZ (B) and hypothalamus (C) of WT and *Ppar*α^−/−^ (KO) mice**. The data were expressed as the media of the number of BrdU+ cells per area (mm^2^) + s.e.m (*N* = 5). Bonferroni's test: ^*^P < 0.05, ^***^P < 0.001 vs. WT-SE group; ^#^*P* < 0.05 vs. KO-SE group; ^$$^*P* < 0.01 vs. WT-EE group. **(D–O)** Representative photomicrographs showing low and high (insets) magnification views of the typical clustering of newborn cells containing the BrdU labeling in the SGZ, SVZ and hypothalamus of WT or KO mice in a standard (SE) or enriched (EE) environment. Scale bars (100 and 20 μm for insets) are included in each image.

Scattered clustering of newborn cells containing nuclear BrdU labeling was observed in the SVZ of the 18 months old mice (Figure 3). Qualitatively, this arrangement of the SVZ BrdU+ cells was more prominent in the mice lacking PPARα receptors. The BrdU+ cells found in the SGZ and the hypothalamus of the older mice was very scarce. Qualitatively, we also observed a higher number of BrdU+ cells in the SGZ and the hypothalamus of the 18 months old mice exposed to the enriched environment (Figure 3).

Two-way ANOVA showed an environmental enrichment effect on the number of BrdU+ cells in the SGZ [*F*_(1, 21)_ = 5.62; *P* < 0.05] and the hypothalamus [*F*_(1, 21)_ = 7.2; *P* < 0.05], but not in the SVZ (Figure 3). A genotype effect (WT and KO) on the number of BrdU+ cells was found in the SVZ [*F*_(1, 21)_ = 48.34; *P* < 0.001] and the SGZ [*F*_(1, 21)_ = 7.72; *P* < 0.05], but not in the hypothalamus. Interaction between environmental enrichment and genotype was detected in SVZ cell proliferation [*F*_(1, 21)_ = 7.47; *P* < 0.05] and hypothalamic cell proliferation [*F*_(1, 21)_ = 4.32; *P* < 0.05], that is, PPARα deficiency produced a different effect on SVZ and hypothalamic cell proliferation in an environment dependent-manner.

A Bonferroni analysis showed that the number of BrdU+ cells increased in the SVZ, but decreased in the SGZ and the hypothalamus of the KO mice compared to the WT ones when they were housed in a standard environment (^*/***^P < 0.05/0.001; (Figures 3A–C,D,F,H,J,L,N). The number of BrdU+ cells was also increased in the SVZ of the KO mice compared to the WT ones when they were housed in an enriched environment for 20 days (^$$^*P* < 0.01; (Figures 3A,E,G). The number of BrdU+ cells in the SVZ of the KO mice housed in an enriched environment decreased compared to those of the respective standard environment KO group (^#^*P* < 0.05; (Figures 3A,F,G). In contrast, an increase in the number of BrdU+ cells was found in the SGZ of the WT and KO mice (Figures 3B,H–K) and the hypothalamus of the KO mice (Figures 3C,N,O) housed in an enriched environment (^*^/^#^*P* < 0.05).

### Effect of age, genotype, and environmental enrichment on SGZ cells expressing doublecortin

**Figure 4:**
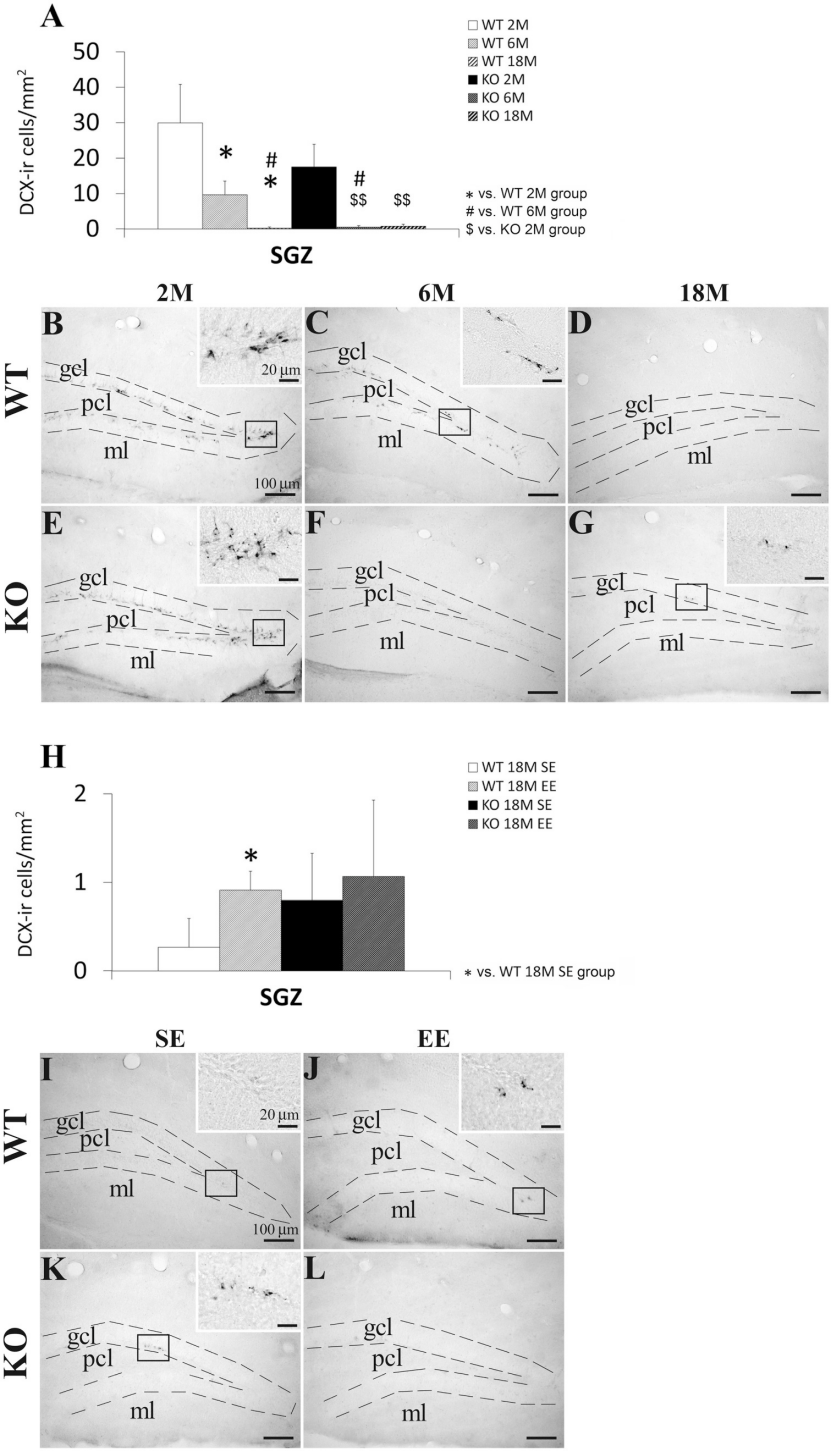
**Effect of age (2, 6, and 18 months) (A–G) and enriched environment (H–L) on the number of cells expressing doublecortin (Dcx) in the SGZ of WT and *Ppar*α^−/−^ (KO) mice**. The data were expressed as the media of the number of Dcx+ cells per area (mm^2^) + s.e.m (*N* = 5). Bonferroni's test: ^*^P < 0.05 vs. WT 2M group or WT 18M SE group; ^#^*P* < 0.05 vs. WT 6M group; ^$$^*P* < 0.01 vs. KO 2M group. **(B–G)** Representative photomicrographs showing low and high (insets) magnification views of the Dcx+ cells in the SGZ of 2, 6, and 18 months old WT and KO mice. **(I–L)** Representative microphotographs showing low and high (insets) magnification views of the Dcx+ cells in the SGZ of 18 months old WT and KO mice in a standard (SE) or enriched (EE) environment. Scale bars (100 μm and 40 μm for insets) are included in each image.

We analyzed the number of Dcx+ cells in the SGZ of the WT and KO mice with 2, 6, and 18 months old, and with or without environmental enrichment (Figure 4). The presence of the Dcx+ cells in the SGZ was more evident in the younger mice that the older mice. Numerous cells expressing Dcx presented a cell body with a pyramidal shape and a main fiber that crossed the granular cell layer (Figures 4B,E).

Two-way ANOVA showed an age effect (2, 6, and 18 months) on the number of Dcx+ cells [*F*_(2, 12)_ = 10.27, *P* = 0.0025]. No genotype effect was observed, but interaction (age vs. genotype) was found when both 6 and 18 months old mice were only considered in the analysis [*F*_(2, 8)_ = 6.03, *P* = 0.0396]. This result suggested that PPARα produced a different effect on the number of SGZ Dcx+ cell from 6 months old onward in a genotype-dependent manner. Two-way ANOVA did not show an interaction and an environmental enrichment or genotype effect on the number of Dcx+ cells in the SGZ of the 18-months-old mice, suggesting that PPARα deficiency did not produce a different effect on the number of SGZ Dcx+ cells in an environment dependent-manner.

After performing the Bonferroni analysis, we found a decreased number of Dcx+ cells in the SGZ of the 6 and 18 months old WT mice compared to that of the 2 months old WT mice (^*^P < 0.05) as well as a decreased number of Dcx+ cells in the SGZ of the 18 months old WT mice compared to that of the 6 months old WT mice (^#^*P* < 0.05; Figures 4A–D). The number of Dcx+ cells was also lower in the SGZ of the 6 and 18 months old KO mice than that of the 2 months old KO mice (^$$^*P* < 0.01; (Figures 4A,E–G). Interestingly, the number of Dcx+ cells decreased in the SGZ of the 6 months old KO mice compared to that of the respective 6 months old WT mice (^$^*P* < 0.05; (Figures 4A,C,F). The Bonferroni analysis also indicated that the number of Dcx+ cells increased in the SGZ of the 18 months old WT mice when they were housed in a enriched environment compared to the WT ones with standard environment (^*^P < 0.05; (Figures 4H,I,J). No change was observed in the 18 months old KO mice with enriched environment (Figures 4H,K,L).

## Discussion

**Figure 5:**
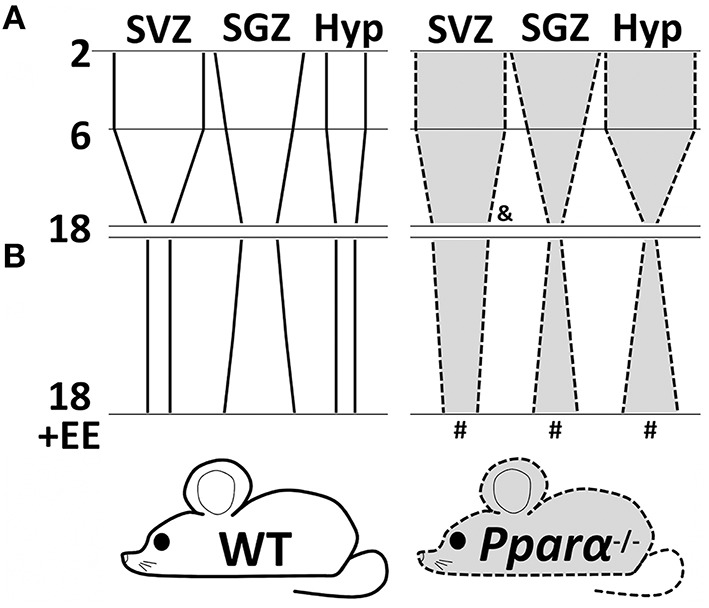
**A schematic diagram summarizing the changes of cell proliferation (BrdU+ cells) in the SVZ, SGZ and hypothalamus of wild-type (continuous lines and white background) and Pparα-/- (dashed lines and gray background) mice during aging (A) and after an exposure to an nutritional and environmental enrichment for 20 days (B)**. Symbols indicate significant changes of cell proliferation between genotypes (WT vs. Pparα^−/−^) in the 18 months old mice (&) and between environments (SE vs. EE) in the 18 months old Pparα^−/−^ mice (#).

In the present study we propose that PPARα may play a differential modulatory role in the maintenance of the adult cell proliferation depending on the neurogenic niche, the age and the environment under our experimental conditions. Our results are consistent with the well-known neurogenesis decline through the mouse lifespan (McDonald and Wojtowicz, 2005; Ngwenya et al., 2015). However, the absence of PPARα alleviated the age-related reduction of NPC proliferation (BrdU+ cells) in the SVZ of the 18 months old mice, while no genotype effect was detected regarding this fact in the SGZ and hypothalamus (Figure 5A). Interestingly, an accentuated reduction in the number of Dcx+ cells was observed in the SGZ of the 6 months old *Ppar*α^−/−^ mice. An enriched environment involving different contextual stimulation consisting of objects (ramps, floor platforms, tunnels, and toys) and treats (fruits, crackers, and cheese) for 20 days in aged mice lacking PPARα counteracted the accentuated decrease of NPC proliferation in the SGZ and the hypothalamus, and reestablished the cell proliferation reduction that was observed in the SVZ of older wild-type mice (Figure 5B). The enriched environment likely increased both cell proliferation and maturation in the hippocampus of the 18 months old WT mice, as the number of BrdU+ cells and Dcx+ cells was elevated in the SGZ. However, the enriched environment did not increase the very low number of the SGZ Dcx+ cells in the 18 months old *Ppar*α^−/−^ mice, as could be expected. In summary, the SGZ of the older *Ppar*α^−/−^ mice exposed to environmental enrichment showed an increase in cell proliferation but no change was found in the number of immature neurons expressing doublecortin. These results in the SGZ suggest an anticipation of the age-dependent neurogenesis decline as a similar low number of Dcx+ cells was observed in the 6 and 18 months old *Ppar*α^−/−^ mice compared to the progressive cell number decline in the respective wild-type mice. A putative impaired neurogenesis in the hippocampus of the older *Ppar*α^−/−^ mice may be of great therapeutic relevance regarding the positive correlation between cognitive dysfunctions and hippocampal neurogenesis decline during aging (Drapeau et al., 2003; Aizawa et al., 2009; Coras et al., 2010). Further studies will be needed to elucidate the specific role of PPARα signaling system (including endogenous activators, such as OEA, and their synthesizing and degrading enzymes) in cell proliferation and neuronal maturation in the hippocampal SGZ of the adult brain during aging.

PPARα and theirs endogenous ligands such as OEA are involved in the modulation of antioxidant responses, neurotransmission, neuroinflammation, and neurogenesis, which can confer protective roles in models of neurodegenerative and neurocognitive diseases (Heneka et al., 2007; Aleshin and Reiser, 2013; Fidaleo et al., 2014). This nuclear receptor is highly expressed in neuronal populations of certain brain areas such as the hippocampus (Moreno et al., 2004; Rivera et al., 2014), which are particularly implicated in the modulation of learning and memory consolidation. An interesting study aimed at correlating PPARα activation and the expression of brain-derived neurotrophic factor (BDNF) in hippocampal neurons demonstrated an improved learning and memory in an animal model of Alzheimer disease via PPARα (Roy et al., 2015). Overall, PPARα activation by endogenous (OEA and palmitoylethanolamide) and/or exogenous (fenofibrate) agonists shows a neuroprotective role by increasing brain cell proliferation and improving neuronal survival associated with spatial long-term memory after different cerebral insults such as whole-brain irradiation, cerebral ischemia, and Alzheimer disease (Ramanan et al., 2009; Scuderi et al., 2012; Yang et al., 2015). In this sense, it is appropriate to evaluate whether PPARα regulates the NPC proliferation decline throughout adulthood. Interestingly, we demonstrate that PPARα likely acts as a differential, homeostatic modulator of aging-induced cell proliferation decline in the principal neurogenic niches of the adult brain. As we have found differences in the rate of NPC proliferation in the SVZ of the older mice lacking PPARα (18 months old), in contrast with the younger mice (2 months), the participation of this receptor in neurogenesis may be directly related to aging. Further studies will be needed to assess the protective role of PPARα associated with improved neurogenesis and better memory performance among the elderly.

Environmental factors can provide profound influences on brain development and functioning during lifespan. It is well-documented the beneficial effects of enriching environments with physical, social and sensory stimuli on neurogenesis, neuronal sprouting, learning, and memory, suggesting an important therapeutic approach in the prevention and/or recovery of neurodegenerative diseases (Mohammed et al., 2002; Kuzumaki et al., 2011; Garthe et al., 2016). Enriched inputs are able to modulate brain plasticity during all stages of life as a consequence of a variety of responses triggered by neurotrophic and neurogenic factors (Pérez-Martín et al., 2005; García-Segura et al., 2007; Arevalo et al., 2015). Regarding the influence of the environment in the old age, a recent study demonstrated that an enriched environment counteracted the decrease of BDNF levels in the hippocampus of molarless mice, which was in turn associated with the amelioration of proliferation, survival and differentiation of newborn cells in the SGZ and the improvement of hippocampus-dependent spatial memory (Kondo et al., 2015). A previous study indicated that the growth hormone may provide a protective role in old animals as its administration ameliorated neuronal loss associated with aging (Azcoitia et al., 2005). In agreement with this study, chronic IGF-1 treatment reduced spatial learning impairment and up-regulated neural proliferation in the SGZ of aged female rats exposed to prenatal stress (Darnaudéry et al., 2006). To go further into the hypothesis, we evaluated the beneficial effects of an enriched environment involving different sets of objects and games (ramps, floor platforms, tunnels and toys) accompanied by nutritional enrichment (fruits, crackers and cheese) for 20 days in old aged mice lacking PPARα that showed impaired effects on NPC proliferation. In agreement with this premise, our results indicated that the enriched environment counteracted the PPARα deficiency-induced reduction of cell proliferation in the SGZ and the hypothalamus of older mice. Surprisingly, PPARα deficiency increased cell proliferation in the SVZ of the older mice. These results should be interpreted regarding a specific vulnerability of *Ppar*α^−/−^ mice associated with senescence and deficiency of PPARα-mediated neuroprotection. Both facts probably resulted in a poor survival of new neurons in those brain regions, such as olfactory bulb and striatum, which are tangentially and radially targeted by the NPC of the SVZ. Thus, it is consistent that vulnerability associated with old age and PPARα deficiency could be the major inducer of striatal neurogenesis. Previous studies established that inflammation accompanying an ischemic insult triggers a marked increase of NPC in the SVZ and leads to the recruitment of formed neuroblasts to repair the damaged striatum (Arvidsson et al., 2002; Thored et al., 2006; Chapman et al., 2015; Lin et al., 2015). Our data evidenced that *Ppar*α^−/−^ mice housed in enriched environment for 20 days showed a partial recovery toward the basal rate of NPC proliferation that characterizes the SVZ of the older wild-type mice. The enriched environment including contextual stimulation (objects and games) and nutritional treats likely contributes to neuroprotection, which can, in turn, enhance survival of the new-born neurons and, as a consequence, the restoration of the NPC proliferative rate to control levels in the SVZ. We hypothesize that this process is closely regulated by neurotrophic factors (e.g., IGF-1 and estradiol; García-Segura et al., 2007; Arevalo et al., 2015). Future studies will be designed to elucidate the regulation of the main neurotrophic factors (BDNF, IGF-1, estradiol) by PPARα activation along the elderly.

It should be highlighted the discrepancies of the effects of the enriched environment on NPC proliferation in the SVZ, SGZ, and hypothalamus of the *Ppar*α^−/−^ mice. A similar discrepancy regarding the different neurogenic zone of the adult rodent brain was also described in previous studies when neurogenesis was evaluated after different insults such as the exposure to cocaine, alcohol, or a very high fat diet (Rivera et al., 2011, 2015a,b; Blanco-Calvo et al., 2014). In previous studies, we described that adult male rats with a prolonged intake of a high fat diet and treated with the CB1 receptor antagonist AM251 or mice with a repeated administration of cocaine and treated with the CB1 and CB2 receptor antagonists Rimonabant and AM630, respectively, showed an increased neural proliferation in the SGZ, but a decreased neural proliferation in the SVZ (Rivera et al., 2011; Blanco-Calvo et al., 2014). In two recent studies from our group, we described that the reduction of neural proliferation in the SVZ and SGZ after the inhibition of the fatty acid amide hydrolase (FAAH) by the repeated administration of URB597, and the increase of neural proliferation in the SVZ and SGZ after CB2 receptor stimulation in alcohol-exposed rats were not observed in the hypothalamus (Rivera et al., 2015a,b).

As a conclusion, the present study indicates that PPARα deficiency differentially alters the age-induced decline of NPC proliferation in relevant neurogenic niches (SVZ, SGZ, and hypothalamus) of the adult mouse brain. These data suggest a potential modulatory role for PPARα in the age-induced neurogenesis decline. This modulation is brain area dependent. While, in the SVZ, the absence of PPARα promotes more proliferation at older ages, in the SGZ the absence of this receptor may lead to maintain a deficiency in neurogenesis. These results suggest that PPARα in the SVZ likely acts as a neuroprotective factor. The exposition to nutritional and environmental enrichments of the older mice (18 months old) reversed the *Ppar*α^−/−^-induced impairment of NPC proliferation in the three neurogenic zones analyzed, but no change was found in hippocampal neuronal maturation. In the SVZ the enhanced cell proliferation is not further potentiated by environmental enrichment, whereas EE boosts cell proliferation in the SGZ and hypothalamus of the *Ppar*α^−/−^ animal. Further studies with PPARα activators such as OEA are needed to investigate whether this role can be of therapeutic value for behavioral impairment associated with aging.

## Author contributions

All authors had full access to all data in the study and take responsibility for the integrity of the data and the accuracy of the data analyses. Study concept and design: MP, PR, EB, FR, and JS. Acquisition of data: PR, EB, CL, and JD. Analysis and interpretation of data: MP, PR, EB, FR, and JS. Drafting of the manuscript: MP, FR, and JS. Critical revision of the manuscript for important intellectual content, obtained funding and study supervision: FP, AS, FR, and JS.

### Conflict of interest statement

The authors declare that the research was conducted in the absence of any commercial or financial relationships that could be construed as a potential conflict of interest.

## Abbreviations

3v: third ventricle
ARC: arcuate nucleus of hypothalamus
BrdU: 5-bromo-2′-deoxyuridine
Dcx: doublecortin
EE: enriched environment
gcl: granular cell layer
lv: lateral ventricle
ml: molecular layer
NPC: neural proliferative cells
pcl: polymorphic cell layer
*Pparα*^−/−^: homozygous knockout in peroxisome proliferator-activated receptor alpha
SE: standard environment
SGZ: subgranular zone of dentate gyrus
Str: striatum
SVZ: subventricular zone of the lateral ventricles
VMH: ventromedial nucleus of hypothalamus
WT: wild-type.
